# The King's Wish

**Published:** 1912-06-01

**Authors:** 


### The Hospital

The Workers' Newspaper of Administrative Medicine and Institutional Life, Administration, National Insurance and Health.

No. 1351, Vol. LII. SATURDAY, JUNE 1, 1912. PRICE ONE PENNY.

### THE KING'S WISH.

The people of London for the most part are no doubt ignorant of the fact that by the King's wish Sunday, June 9, should go down to history as memorable for all time. The people of these islands have come to a stage in their history where the individual suffers more from excess in all things than ever before—excess of sentiment, which sadly hampers good government; excess of hurry, due to the attempt to crowd too much into too limited a time; excess of so-called sport, mainly attendance at centres where a few members of the race compete with the highest skill for a prize; excess of strain from unwisdom or ignorance in overdoing necessary exercise; excess of competition in dress, in obtaining the best places, in pushing the individual forward. In short, excess in everything, everywhere, on the part of ever-increasing numbers of the population, threatens the character of the nation, and for its security a halt must be called. What England needs in these days is quietness and confidence, and more and more the enforcement upon the individual of a standard of life; more simplicity in dress, in living, in amusements, in work, in everything. We want some annealing power amongst us to quicken and re-establish family life. At its best a great nation is at bottom so unitedly patriotic as to constitute in fact one great family.

Holding these views and regarding them as of paramount importance to the health and happiness, the strength and permanence of the English nation, we welcome with a special thankfulness the expression of the King's wish in regard to Hospital Sunday, 1912. St. Paul's Cathedral has been the centre and scene of many notable services, national and religious. Under the late Dean, St. Paul's has become more and more the great civic church of the Metropolis of the Empire, where all classes of the community have acquired the habit in increasing numbers of resorting periodically to offer praise, worship, and thanksgiving. Many times have successive monarchs attended at St. Paul's to render thanksgiving to God for their recovery from illness or for victory over the nation's foes. Often have the people crowded St. Paul's on memorable festivals with grateful and full hearts. But the service on June 9, 1912, Hospital Sunday, will by the King's wish be essentially the People's Day, when all classes of the community, taking their lead from St. Paul's Cathedral, where the King will attend privately with the Queen, may make a point of being present in the House of God which they habitually attend in order that they may join with their Majesties in showing practical sympathy with the sick, and thanksgiving for the blessings of health which individually they may enjoy.

The service at St. Paul's Cathedral on the 9th inst., like the service on that day in every place of worship of every denomination throughout London, will be free from all ceremonial, so that everybody everywhere will be free to join in the service and add their voice to the harmony of praise for the blessings all enjoy. There are to be no tickets, no railed-off spaces, no processions; but the King and Queen will attend quite privately at the Cathedral of the Metropolis of the Empire, where, in all simplicity, humility and heartfelt devotion, they will join as man and woman with every other man and woman who has imagination and heart, on this great day of thanksgiving, when all the sects join hands in the cause of the sick. Hospital Sunday during the last decade seems somehow to have lost its hold upon the clergy and ministers of religion, who have let its lessons and great opportunities grow dimmer and dimmer in the memory of the people. This spontaneous act of King George and Queen Mary to go quietly together to the Cathedral Church, as the men, women, and children in Birmingham used to go in the old days when Hospital Sunday was a living reality there, will, we hope, touch the hearts and consciences of bishops, clergy, and people, so that every family in London may this year become fully conscious that June 9 is Hospital Sunday, and that that day will afford them a special opportunity for worship and thanksgiving which should appeal to them with irresistible force. Nothing in this life is really worth doing that does not entail the exercise of will-power and deliberate choice on the part of the individual. No doubt, habit is increasingly making Sunday in this country less and less a day of worship, and more and more a day of pleasure. That is why this wish of King George, and his method of expressing it, should go straight to the hearts and minds of Londoners of both sexes and all classes. If it does, the congregations in all the churches and places of worship within the metropolitan area next Sunday week will be the greatest on record. We make bold to say and to think that enjoyment, happiness, and all that is best in life will be given in fuller measure to those who follow the example of His Majesty the King on that day than to those who disregard his example and by deliberate choice devote their whole energies and time to the pursuit of pleasure. The true royalty of human life is its capacity for compassion. To what extent the inhabitants of modern London possess this great quality the attendance in places of worship on Hospital Sunday, June 9, will show.

